# Novel contactless approach for assessment of venous occlusion plethysmography by video recordings at the green illumination

**DOI:** 10.1038/s41598-017-00552-7

**Published:** 2017-03-28

**Authors:** Alexei A. Kamshilin, Valeriy V. Zaytsev, Oleg V. Mamontov

**Affiliations:** 1 Department of Computer Photonics and Videomatics, ITMO University, St. Petersburg, 197101 Russia; 2 Federal Almazov North-West Medical Research Centre, Department of Circulation Physiology, St. Petersburg, 197341 Russia

## Abstract

Occlusion plethysmography is an important method for assessment of the status of the cardiovascular system, which provides valuable information concerning arterial and venous blood flow, including mechanisms of their regulation. All up-to-date systems estimate change of the limb’s volume during occlusion by contact-type sensors. The objective of the research is demonstration of feasibility of the novel approach to measuring the blood flow during venous occlusion by using imaging photoplethysmography (PPG). Twenty healthy individuals participated in the experiment. We used four synchronized video cameras to record all-around view of the forearm illuminated by the green light. After the recording, the PPG waveform was calculated in more than 4000 non-overlapping Regions of Interest (ROI). In the most of the ROIs, the waveform shape was typical for classical plethysmography with the distinctive linear growth of the signal. Speed of the signal change was the same along the forearm but it varied along the forearm’s circumference. These findings allows us to hypothesize that the PPG waveform is the direct consequence of the forearm blood flow. Therefore, the novel technique could be applied to the same medical examinations as the classical plethysmography, but it is more advantageous because of non-contact nature and easiness in implementation.

## Introduction

Venous occlusion plethysmography is an important physiological manoeuvre to examine the peripheral blood flow and peripheral resistance which is changing in remodeling arteries. In this manoeuvre, return of venous blood is briefly interrupted for interval of 4–10 s by rapid inflation of a brachial cuff allowing arterial blood inflow into the limb^1^. The rate of blood flow is estimated by measuring limb volume change during the manoeuvre. Such a measurement is challenging task since small changes of complicated limb geometry caused by the blood inflow coexist with other accidental variations of the limb parameters (e.g., due to muscles constrictions/relaxations). Moreover, it should be carried out with minimal influence on blood vessels. Initially, water and air filled jackets were used for plethysmographic measurements but they were difficult in implementation requiring special skills from the operator^2^. Currently, mercury-in-rubber (or silastic) strain gauge, which measures changes of limb circumference, is widely used technology for monitoring the venous occlusion^1, 3^ in spite of mercury toxicity and problems with incorrect readings, which may appear due to inevitable contact with the skin.

Applicability of non-invasive optical technology referred to as photoplethysmography (PPG) for assessment of venous blood flow was examined, as well^4–6^. Common opinion is that PPG does not yield quantitative information about the blood flow but it is highly sensitive to the flow change^4, 5^. However, previous studies of PPG in applications to venous occlusion monitoring were carried out with contact-type sensors operating with the infrared (IR) light and measuring the blood-flow parameters in a single point^4–7^. Choice of the IR light for tissue illumination is a consequence of the commonly accepted model of light interaction with living tissue behind the photoplethysmography. According to this model, the PPG waveform mainly originates from the relative changes of blood volume in major blood vessels, which modulate the light absorption in the tissue^7–10^. IR light is considered to possess enough penetration depth into the tissue for interaction with these vessels. However, the source of the PPG signal has been the subject of continuing debate^10^. The main problem of the conventional PPG model is that it can hardly explain recent observations obtained with imaging photoplethysmography (IPPG) systems^11–13^ where the largest signal at the heartbeat frequency was observed at the green light in spite of its smaller penetration into the tissue and impossibility interact with large blood vessels.

Recently, an alternative explanation of the PPG-waveform origin was proposed in our group^14^. New model hypothesizes that the green light is modulated mainly due to compression/decompression of the capillary bed occurring synchronously with changes of blood pressure in vessels located in the place of the measurement. It is also considered that dermis contains both blood and lymphatic capillaries which are incompressible and do not pulsate at the heartbeat rate^7^ but the distance between adjacent capillaries can be readily changed^15^ resulting in modulation of their density and, consequently, of the blood volume interacting with the slightly penetrating green light. In other words, the PPG signal negatively correlates with the blood pressure applied to the capillary bed (increase of the pressure lead to decrease of the remitted light intensity)^14^. If the new PPG model is correct, one should be able to monitor changes of the venous pressure in the course of venous occlusion by using IPPG system.

In this paper, we present the experimental observations showing feasibility of assessment the venous occlusion plethysmography by means of IPPG system with simultaneous video recording of a forearm by four video cameras at green illumination.

## Results

### Cameras response on venous occlusion

**Figure 1:**
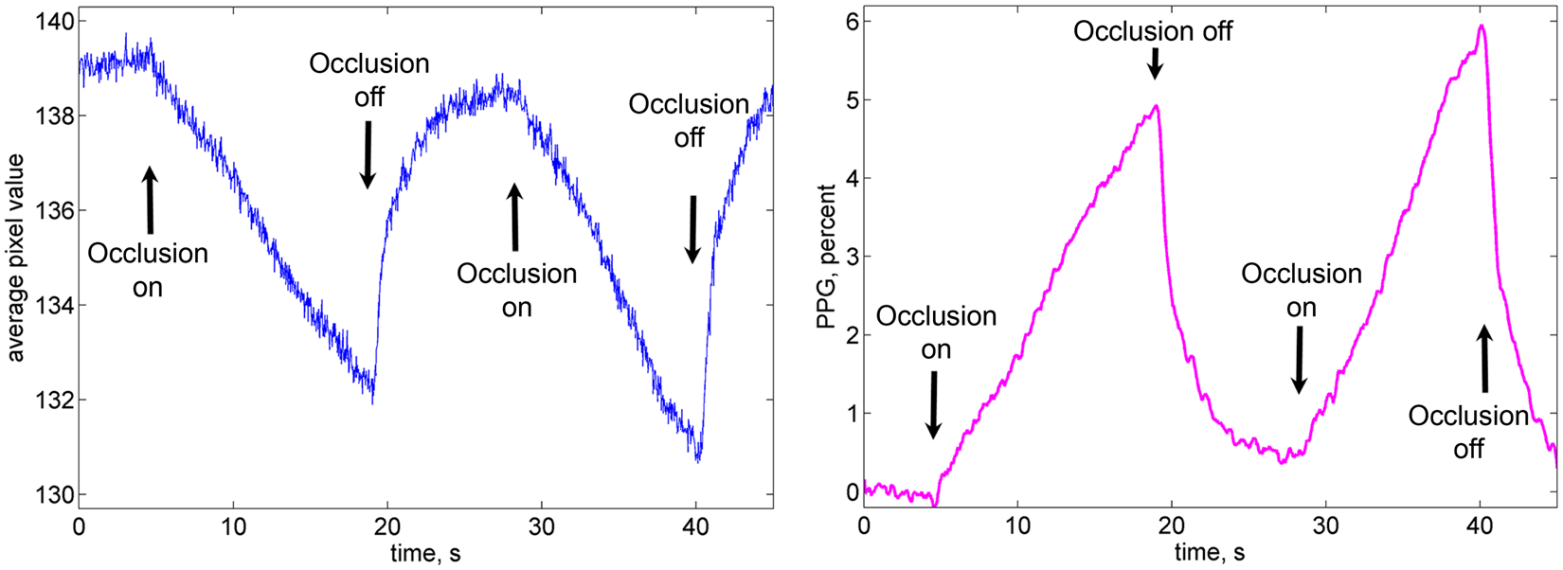
Typical PPG waveform. (**a**) Evolution of the mean pixel value in an arbitrary chosen ROI in the forearm during double venous occlusion. (**b**) The respective PPG waveform after its normalization to the mean pixel value measured before the first occlusion, deduction of the unity, and inversion.

In our experiments, four videos of a subject’s forearm were synchronously recorded during 45 s providing all-around view of the forearm. After fifth second of the recording, the brachial cuff was rapidly inflated up to pressure of 40 mmHg interrupting the return of venous blood for an interval of 10 s. Then the cuff was deflated for 10 s following by another venous occlusion. All the recorded data were processed offline. In each view of the forearm, we selected small Regions of Interest (ROIs) to cover side-by-side the central part of the forearm. Size of the ROI was 5 × 5 pixels in the video frame or 1.7 × 1.7 mm^2^ at the forearm. Typical frame-by-frame evolution of the mean pixel value in an arbitrarily selected ROI is shown in Fig. 1a. One can clearly see the linear drop of the signal immediately after beginning of the cuff inflation with the restoration to the higher level after the cuff deflation. We interpret this drop by diminishing of the remitted light intensity due to increased light absorption caused by growing blood volume after beginning of the venous occlusion.

To distinguish the blood-volume related changes of the light intensity from possible motion artefacts we carried out preliminary calibration measurements of the camera response on the displacement of an inanimate object. Approaching the object to the camera by 5 mm leads to the increase of the mean pixel value by 3.5 gradations (from 256). Diminishing of the pixel value was observed only in the case of displacing the object outwards the camera. Since venous occlusion leads to increase of the blood volume in the forearm, an expected displacement of the skin caused by occlusion is surely towards the camera lens. Notably, that such a displacement is in the order of tens of micrometers only^16^. Therefore, would it be the camera response on the skin displacement of 20 micrometers, one should observe small increase of the pixel value (about 0.01 gradation) after the cuff inflation. In contrast, it is seen in Fig. 1a that the mean pixel value decreases up to 6 gradations because of diminishing of the remitted light intensity when the brachial cuff is inflated and it immediately increases after occlusion is off. Similar curves were observed with four video cameras installed around the forearm.

To compensate non-uniformity of the incidence light intensity, we normalized the curve to the mean pixel value measured before the first occlusion. For convenience, this curve was inverted to show the positive sign of the blood volume increase because changes of the light intensity detected by the camera sensor are inversely proportional to the blood volume changes^9, 14^. The resulted curve referred to as a PPG waveform is shown in Fig. 1b. It is worth noting that the waveform shown in Fig. 1b has similar features as a typical curve for venous occlusion measured in the method of air plethysmography^1, 16, 17^. These features are following.

- Linear growth of the signal immediately after the initial moment occlusion, which corresponds to the blood-volume increase in the classical plethysmography.
- After the occlusion is off, the signal returns to its initial value during the similar time interval.
- The shape of the signal is reproduced in the series of several consecutive occlusions.

The linear growth of the PPG signal in time after beginning the occlusion was observed in all 20 volunteers participated in our experiments.

### Distorted PPG waveforms

**Figure 2:**
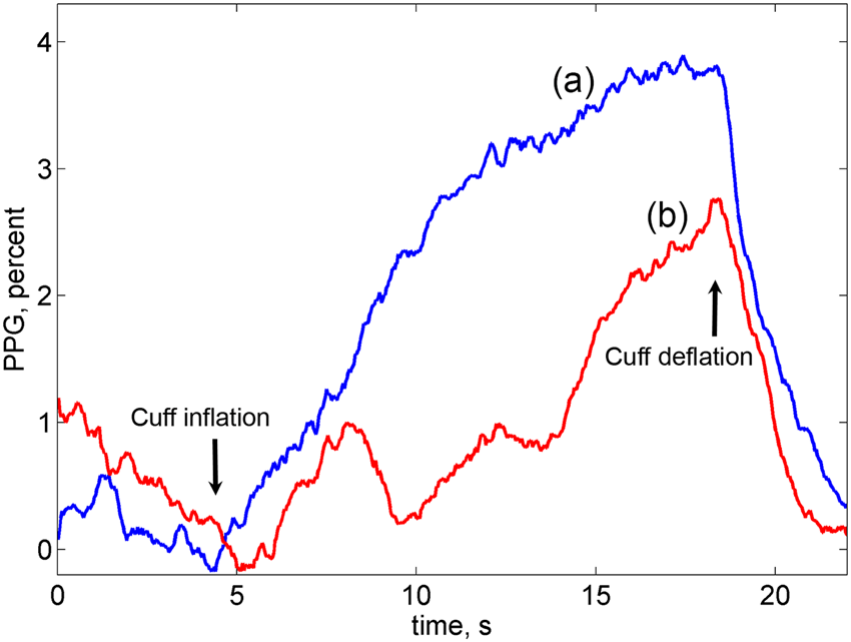
Representative records of two PPG waveforms calculated from the adjacent ROIs. (**a**) Waveform in the ROI identified as a “good”; (**b**) distorted waveform in the untrustworthy ROI.

Since all PPG waveforms in the series of the chosen ROIs are calculated from the same video frames, they represent simultaneously occurring processes in the forearm’s dermis. The linear growth of the PPG waveform during the venous occlusion (see Fig. 1b) was observed in the majority of the chosen ROIs. However, the part of PPG waveforms after the initial moment of the occlusion was distorted in some ROIs. Example of the waveforms instability is shown in Fig. 2. It is worth noting that the waveforms in Fig. 2a and b were calculated in the adjacent ROIs separated one from another by only 1.7 mm. In our following calculations, any waveform in which the initial part does not follow the linear growth during first four seconds (an example shown in Fig. 2b) was considered as distorted, and the respective ROI was marked us untrustworthy. All remaining ROIs were marked as “good”. Possible reasons of the PPG-distortions occurrence will be discussed in the next Section.

**Figure 3:**
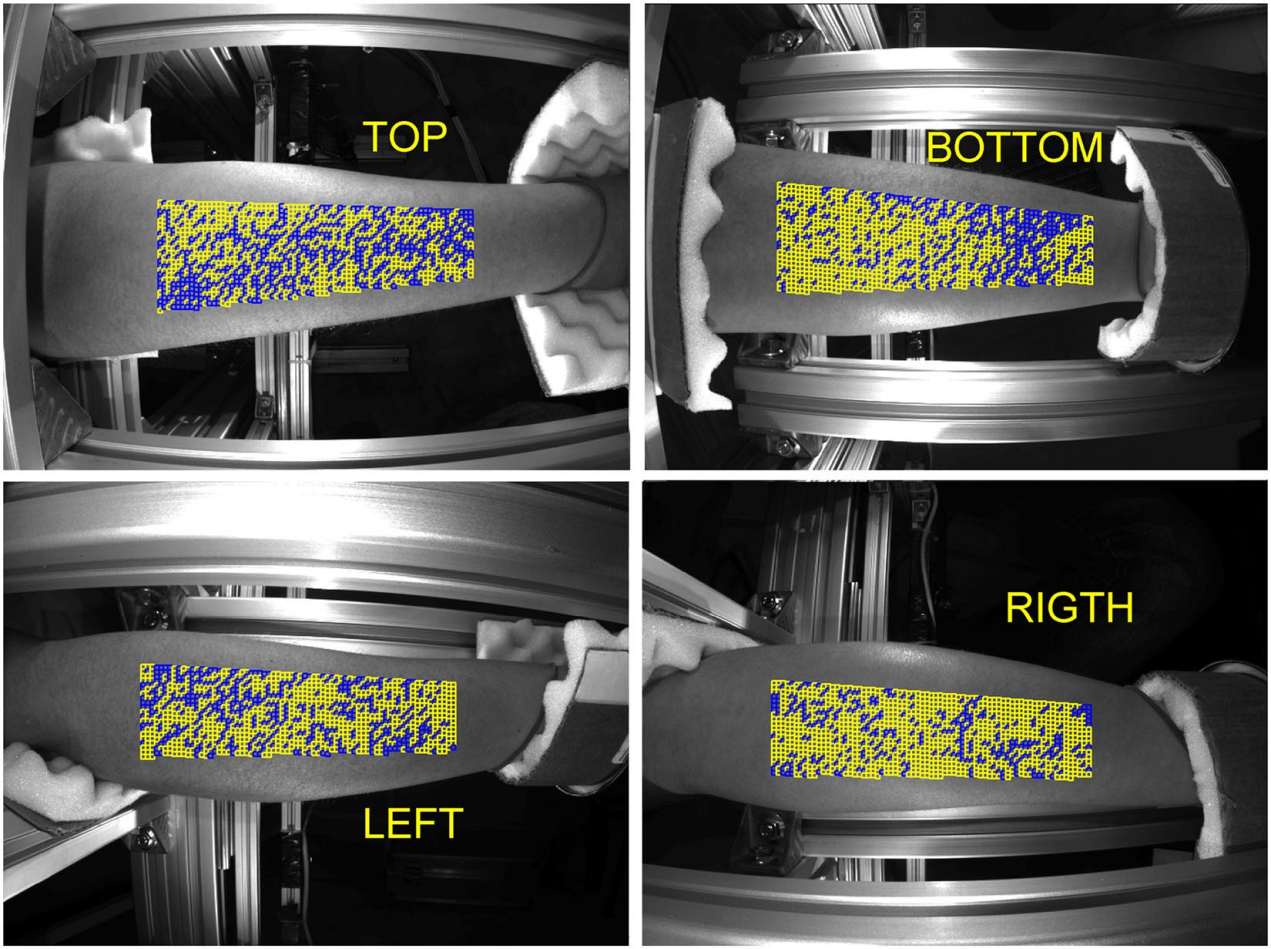
Spatial distribution of the good ROIs in four views of the forehand. Yellow and blue squares show positions of the ROIs with linear and distorted PPG waveforms, respectively.

Figure 3 shows an example of the spatial distribution of the found “good” ROIs in four views of the forearm where the good ROIs are marked by yellow whereas the untrustworthy ROIs are in blue. Both the position and total number of the distorted ROI varies from one subject to another. Nevertheless, large number of “good” ROIs was found for each studied subject: the mean percentage of these ROIs varied from 28% to 95%. Considering that PPG was calculated in more than 4000 non-overlapping ROIs, the minimal number of “good” ROIs (1200) was large enough for the statistical analysis.

**Figure 4:**
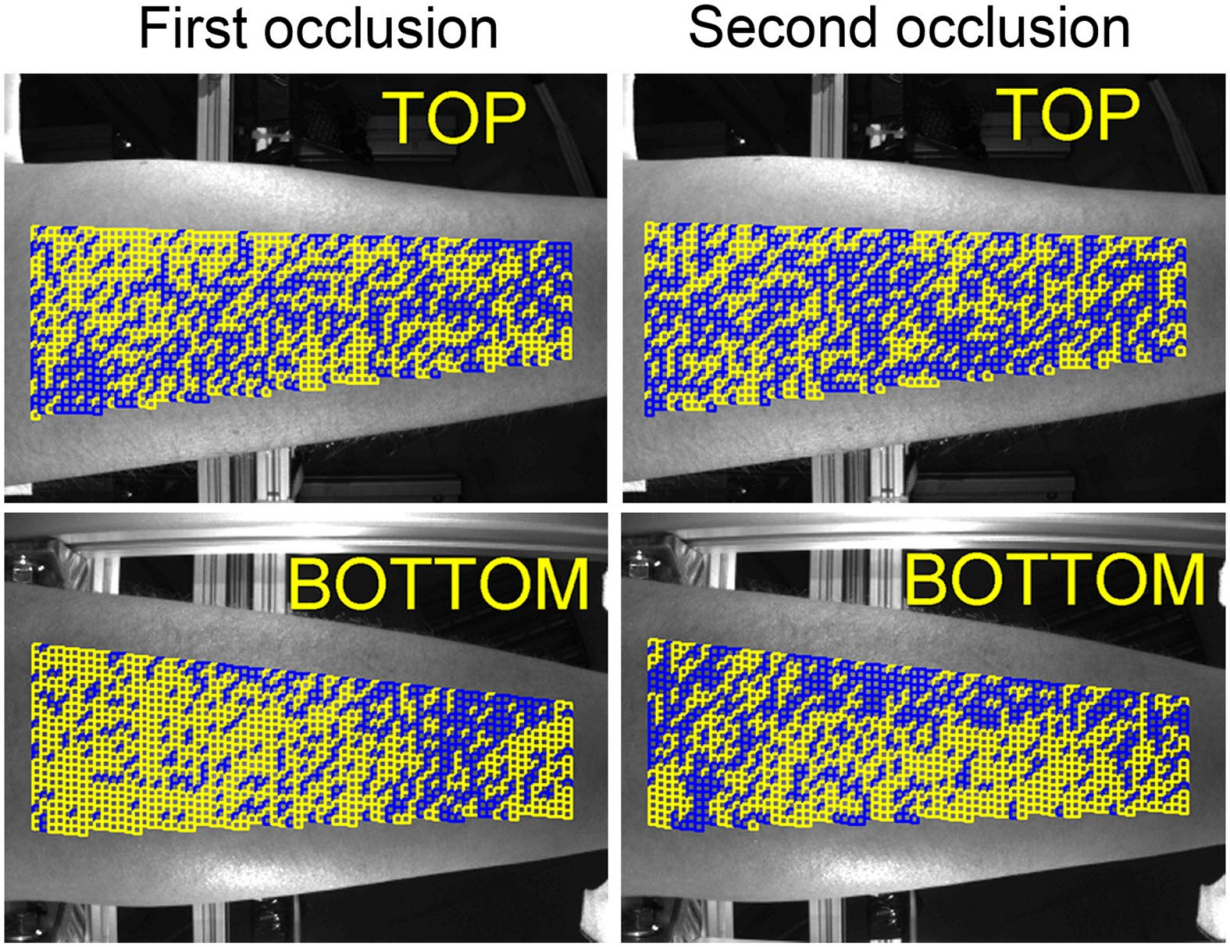
Mapping of the good ROIs distribution. Images in the left column calculated during the first occlusion whereas those in the right column are in the second occlusion that started 10 s later the cuff was deflated. Yellow and blue squares show ROIs with the linear and distorted PPG waveforms, respectively.

It is worth noting that the spatial distribution of ROIs with distorted PPG waveform in the first occlusion does not coincide with that in the second occlusion even so the time difference between these events was less than a half of minute. An example of variability of the “good” ROI position is shown in Fig. 4 in which the left pair of images shows the distribution in the first occlusion, whereas the right pair is for the second occlusion. One can clearly see that the distributions shown in the left and right images are different.

### Rate of PPG-waveform increase

**Figure 5:**
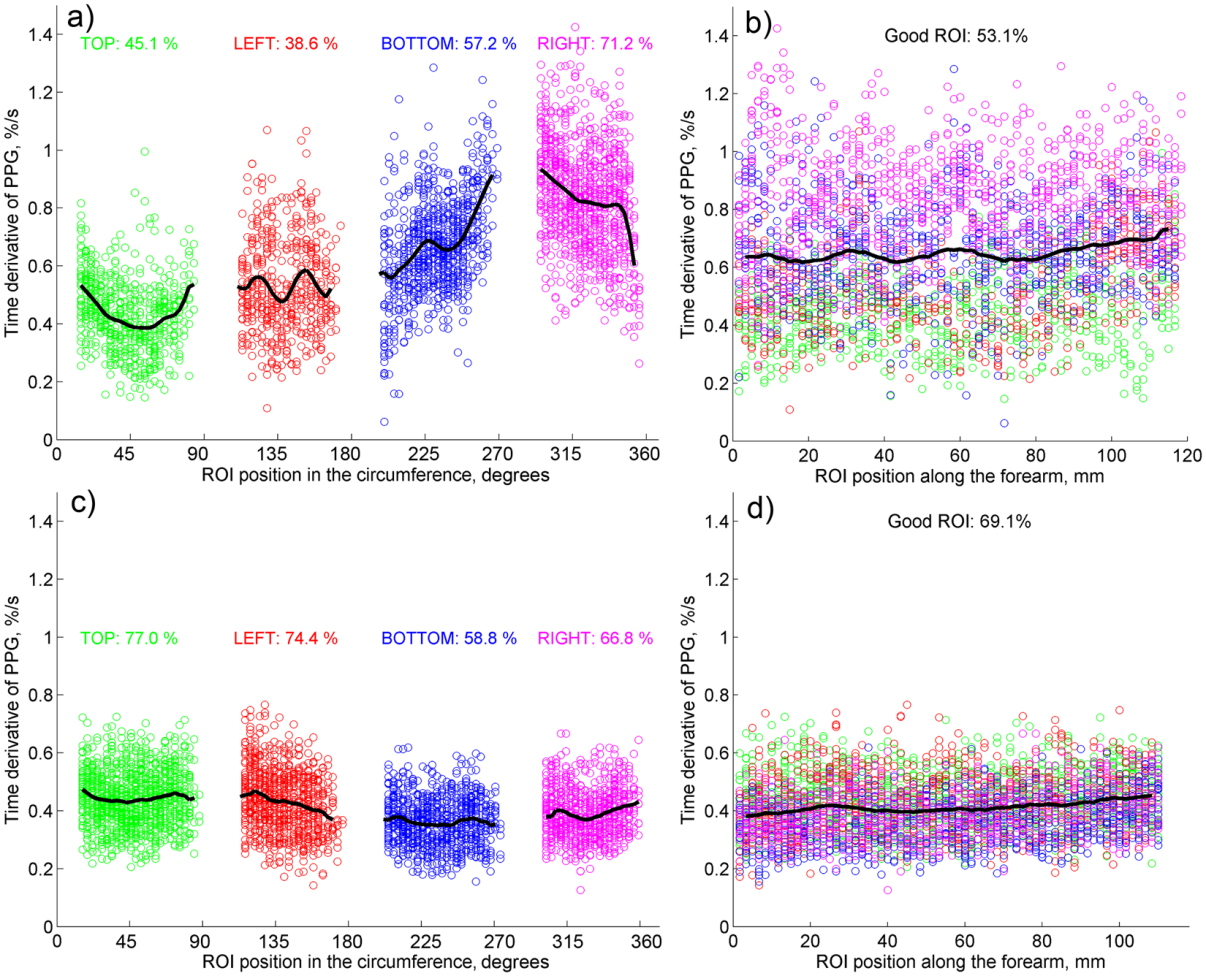
PPG speed calculated for two subjects in “good” ROIs. (**a**) Data scatter as a function of the ROI position along the forearm’s circumference, and (**b**) along the longitudinal direction for one of the subjects. (**c** and **d**) are similar data scatter for another subject. Points measured in different views of the forearm are marked by green, red, blue, and magenta for top, left, bottom, and right cameras, respectively. Colored numbers show the percentage of the good ROIs in the respective view of the forearm.

In the classical plethysmography, the gradient of the signal variations after the beginning of the occlusion represents the speed of the arterial blood inflow^1, 16, 17^. Considering similarity of air-plethysmography and PPG signals, we calculate the time derivative of the PPG waveform (denoted further as a PPG speed) to characterize the system response on the venous occlusion. Representative examples of the PPG-speed data (in percent per second) for two of the subjects is shown in Fig. 5. While the data in Fig. 5a and c were sorted to show dependence of the PPG speed on the ROI position alongside the forearm’s circumference, its dependence alongside the longitudinal direction of the forearm is shown in Fig. 5b and d. In the latter graphs, the abscissa zero is set closer to the elbow. Black solid lines in Fig. 5 show the averaged data in each direction. One can see that the mean PPG speed is almost independent from the position alongside the longitudinal direction of the forearm. No dependence (within the error calculated with the confidence level of 95%) of the PPG speed on the ROI position in the longitudinal direction was found in all 20 studied subjects. In contrast, the PPG speed exhibits clear dependence on the ROI position along the circumference as seen in Fig. 5a and c. The grade of the PPG-speed circular unevenness estimated as the ratio of the difference between the maximal and minimal values to the mean value varies from 30% to 110%. Figure 5a shows the graph with maximal unevenness, whereas Fig. 5c is that with minimal unevenness observed among studied subjects.

The mean PPG speed calculated by averaging the whole set of data in all-around view of the forearm varies from 0.15 ± 0.03 to 0.65 ± 0.13%/s. No dependency of the mean PPG speed on either age or sex of subjects was found.

## Discussion

This research is a first step to development of new technique of quantitative measurements of peripheral blood flow by using occlusion plethysmography. Assessment of the physiological parameters of peripheral circulation (such as volumetric blood flow velocity and peripheral vascular resistance) is very important in clinical diagnostics of cardiovascular diseases^18, 19^, in investigation of neurogenic mechanism of circulation regulation in different diseases^20, 21^, and for estimation of vasomotor reaction on various cures^22, 23^. The proposed method is more advantageous compared with conventional techniques because of its contactless nature of measurements. This allows avoidance of assessment distortions caused by a mechanical impact on the limb under study. These distortions are considered as spurious in contemporary techniques of the occlusive plethysmography^24^. Moreover, the contactless type of measurements opens opportunity to use the same system for studying the limbs’ segments of different sizes, and to increase the system lifetime as a result of nonexploitation of sensors susceptible to a mechanical wear.

In the proposed technique, the event of venous occlusion is monitored by using conventional video recording of a limb under green illumination. In contrast to classical plethysmography, which measures change of the limb’s volume caused by arterial blood inflow, we hypothesize that our technique indirectly measures changes of the blood pressure in big vessels. In normal conditions, outer atmospheric pressure is compensated by the internal pressure in the limb. When venous occlusion starts, inflow of arterial blood increases the internal pressure resulting in enlargement of the limb diameter thus stretching the skin. As we pointed out in the previous Section, expected change of the limb’s diameter due to the venous occlusion is not enough to change the light intensity in the camera sensor of our optical setup. Note that increase of the limb’s diameter should lead to increase of the light intensity registered by all four cameras. In contrast, we observed the linear decrease of the intensity as shown in Fig. 2a. Therefore, we conclude that it is the light absorption inside the skin, which grows up because of the venous occlusion.

Reminding that our experiments were carried out using green light, which penetrates only into superficial layer of the dermis (0.3–0.8 mm)^25^, we suppose very small probability of light interaction with deeply situated blood vessels. Therefore, the most likely reason of the light absorption increase is a rise of the blood volume in the capillary bed. However, the capillaries themselves are not expandable^7, 26^, whereas the connective tissue in the capillary bed is very sensitive to a compression^15^. Consequently, we explain the observed linear decrease of remitted light intensity in accordance with the alternative PPG model^14^: the skin stretching caused by venous occlusion leads to increase of the capillary density, thus increasing both the light absorption and scattering. It is worth noting that recent direct comparison of PPG signals obtained at the wavelengths of 530 and 810 nm showed that an essential part of remitted IR light (λ = 810 nm) is modulated in time due to elastic deformations of dermis caused by variable blood pressure in the arteries^27^. However, the light-modulation amplitude at green light was in average 2.5 times higher than at IR light^27^. To check the influence of the illuminating wavelength on the PPG signal in our experimental arrangement, few videos of a forearm during venous occlusion for different subjects were recorded under IR illumination (810 nm). Like at green illumination, we observed the similar linear decrease of IR-light intensity just after occlusion beginning. However, the relative change of the light intensity caused by the venous occlusion was two – three times smaller than at green light.

Nevertheless, not only growing venous pressure caused by the venous occlusion might affect the grade of the skin stretch. Such physiological processes as muscle contractions, lymphatic flow, thermoregulation, etc. could also vary the stresses locally applied to the skin. We hypothesize that these random processes result in distortions of the PPG waveform shown by the red curve in Fig. 3. Fortunately, IPPG system provides the large number of statistically independent ROIs (4000–4600 in all views of a subject’s forehand) in which the PPG waveform is simultaneously measured. It allows us to select the ROIs with the linear growth of the PPG waveform after the beginning of the venous occlusion as the less distorted by other physiological processes. Observation of independency of the PPG-waveform derivative on the ROI position along the longitudinal direction of the limb (which was found in all 20 studied subjects with an example shown in Fig. 5b) points out the cause-effect relation between the venous occlusion and linear growth of the PPG signal. The hypothesis of stochastic local influence of other physiological processes is supported by the fact that the spatial distribution of “good” ROIs is strongly uncorrelated between two consecutive events of occlusion (see Fig. 4). Variability of the PPG-waveform derivative along the circumference direction (see Fig. 5a) can be explained by topological peculiarities of the blood flow in a particular subject caused by volumetric variations of subjacent muscular tissue or by specific distribution of depository vessels^28^.

However, our experiments do not provide solid proofs that the linear growth of the PPG signal is the direct consequence of the blood accumulation in big veins, which changes the limbs’ volume during venous occlusion. In spite of evident similarity of the PPG signal (Fig. 1b) with classical occlusion curves (see Fig. 2 in ref. 1 or Fig. 4 in ref. 16), we could not completely exclude an explanation of the observed shape of the PPG signal by blood accumulation in the venules, which are close to the skin. To solve this dilemma, an extended experiment aimed to compare influence of global hypothermia on the mean PPG-speed during venous occlusion was carried out with five volunteers. First, two events of venous occlusion of the subject at rest were recorded during 45 s by the IPPG system as it is described in the Sect. Methods. Then a cold ice pack was applied to the chest area of the subject, and another venous occlusion started after 40 s of cold stress induction. It was found that the mean PPG-speed is decreasing by 36 ± 9% (*p* < 0.001) during cold stress, which is consistent with the data obtained by using air plethysmography^29^. Investigation of the skin and muscular vessels reaction on differential provocations is very useful for deeper understanding of the PPG signal origin. For example, local change of the skin temperature results in local change of blood flow in the dermis^30^ affecting the PPG signal^31^, whereas global hypothermia affects the blood flow in muscular vessels, which maximally contribute to the volumetric blood flow of the forearm^32^. In our extended experiment, the skin temperature was the same. Therefore, observed changes of the mean PPG speed during the cold stress unambiguously demonstrate that IPPG system operating with green low-penetrating light is capable of measuring the blood flow in muscular vessels. This extended experiment validates applicability of the IPPG system in assessment of the venous occlusion parameters.

In our data processing, we used a simple criterion of the PPG-waveform linearity for separation of the ROIs with distorted signals. However, it is not evident that this criterion was optimally chosen. Further research is required to determine unified approach of the “good” ROIs selection. It should be noted, nevertheless, that similar problem arises in the classical plethysmography, as well^16^. We believe that such a problem could be solved considering dominating number of the “good” ROIs.

The proposed method in the existing stage can be used for experimental study of the cardiac system responsiveness on various external actions. However, it is not yet applicable for quantitative estimation of the volumetric blood flow, which requires transformation of the PPG rate (in percent per second) into the blood volumetric units. Search of the appropriate calibration technique is the most important next step in the development of new noncontact IPPG system for assessment of the occlusion plethysmography. Assuming correctness of the alternative model of the PPG-signal formation^14^, we think that an adequate calibration could be achieved by pressing a small area of the skin in the limb for the controlled distance of few tens of micrometers during short time before each occlusion event with simultaneous video recording of the limb. This pressing will simulate the skin stretching by the calibrated value, thus resulting in increase of the capillary density, which can be measured by the IPPG system. Such a pressing could be implemented with a glass plate driven by an electromechanical transducer. We have carried out preliminary experiments with the glass plate moving at the constant speed and producing outer pressure on the subject’s skin. It was found that the light intensity remitted from the limb’s area covered by the glass is linearly decreasing with the time, which again confirms the alternative model of the PPG signal formation^14^. Relevant research aimed to demonstrate feasibility to calibrate the proposed system for measuring the blood flow in liters per minute is planning to be carried out in the second step of the system development.

## Methods

### Participants

Measurements were carried out with 20 healthy volunteers (14 men and 6 women). Age of the subjects was varying from 22 to 65 years. All subjects gave their informed consent of participation in the experiment in the written form. The study was conducted in accordance with the ethical standards laid down in the 1964 Declaration of Helsinki. The study plan was approved on February 6, 2016 (Record No. 30) by the research ethical committee of the Federal Almazov North-West Medical Research Center prior the experiments.

### Measurement system

**Figure 6:**
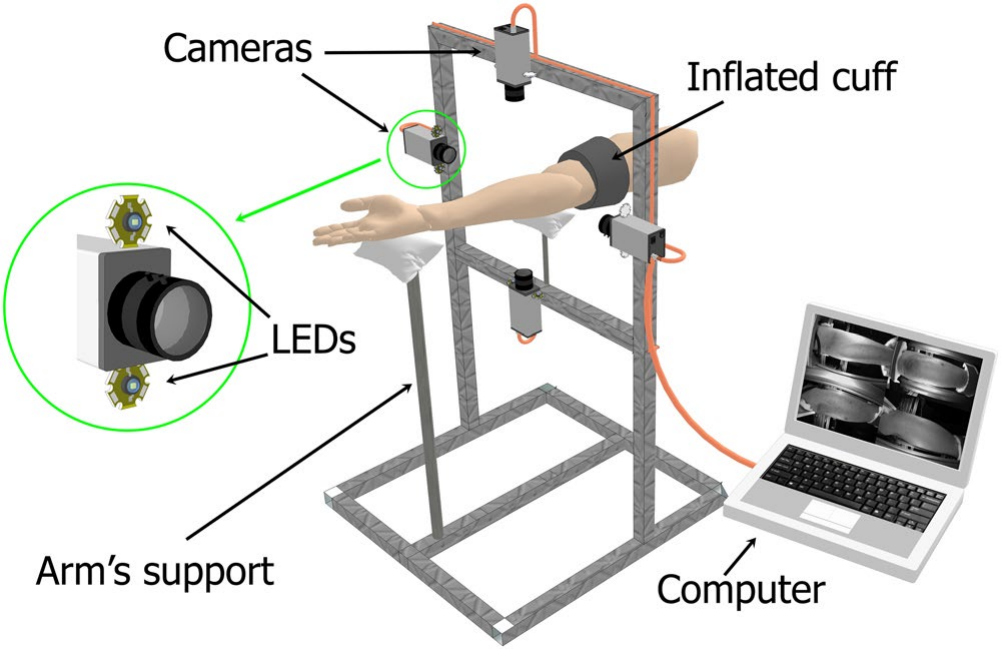
Schematic view of the experimental setup for recording of all-around view of the subject’s forearm during occlusion experiment. Each LED with the output power of 5 W operates at the wavelength of 525 nm (spectral bandwidth 60 nm).

The experimental setup for recording the video frames of the subject was implemented in the reflection geometry as shown in Fig. 6. We used a custom-made IPPG system consisted of four digital cameras of Smartek Vision GC1391MP with the ½-inch photosensitive matrix of Sony ICX267. The cameras were installed around the forearm at the distance of 35 cm to provide all-around video recordings of the forearm. Images in each view of the forehand were focused manually on the camera matrix by the KOWA LM5NCL lenses. Each lens provided the angle of view equal to 79° and 59.4° in the horizontal and vertical plane, respectively. Video frames with the size of 696 × 520 pixels were recorded at the frame rate of 30 frames per second. Two light-emitting diodes (LED) of BL-HP20APGCL-5W STAR operating at the wavelength of 525 nm (spectral bandwidth 60 nm) with the output power up to 5 W were installed nearby each camera lens as shown in the left insert in Fig. 6. The LEDs provided almost uniform illumination in each respective view of the forearm. The output power of the LEDs, the lens aperture, and the sensor exposure were set to exclude overexposed areas in the focused image of the forehand. Intensity of the forehand illumination by the LEDs exceeded the whole background illumination by at least 50 times. The direction of observation provided by the video systems made a small angle with the direction of illumination. All four cameras were connected to a personal computer via the Local Area Network ports. The cameras operated in the waiting mode. Trigger pulse generated in the computer was sent in parallel to all cameras providing simultaneous start of image frames capturing. The images were recorded directly into the Random Access Memory of the computer using Ethernet interface in the portable network graphics (PNG) format. All measurements were carried out in a laboratory maintained at a temperature of 22–24 °C.

### Experimental procedure

During the measurements, the subject was in a comfortable supine position with his hand in the same level of the heart. Both his palm and elbow were on the soft supports. An occlusion cuff was attached to a bottom third of the subject’s upper arm. During the video recording, the subject was asked to avoid any movement of his arm and keep breathing normally. In the fifth second of the recoding, we rapidly (within one second) increased the air pressure in the cuff up to 40 mmHg, maintaining this pressure during next ten seconds. Thereafter, the cuff was rapidly deflated, and no pressure was applied during more ten seconds. Then the same sequence of the cuff inflation/deflation was applied again. Duration of the experiment was 45 seconds in which we recorded four series of 1350 frames.

### Data processing

All recorded video frames from four cameras were processed off-line by using custom software implemented in the MATLAB platform. First, we selected an area for analysis (sizing approximately 110 × 35 mm^2^ in the central part of each view of the forearm to exclude boundary areas in which the illumination angle is rather high because of the rounded forearm’s shape. Second, we covered the selected areas by small ROIs side-by-side with size of 5 × 5 pixels, which corresponds to the area of 1.7 × 1.7 mm^2^ at the forearm’s skin. In each ROI we calculated frame-by-frame evolution of the mean pixel intensity. Typical example of this evolution is shown in Fig. 1a. Non-uniformity of the skin illumination was compensated by normalization of the obtained curve to the mean pixel intensity measured just before the occlusion moment. After deducing the unity and inverting the sign, we calculated the PPG waveform, which positively correlates with the changes of the blood pressure. Such a waveform is shown in Fig. 1b with the specific linear growth of the signal after the start moment of the occlusion.

To identify the distorted waveforms we applied the criterion of the signal linearity. A waveform was considered as distorted if its mean derivative calculated during first two seconds of the occlusion make an angle larger than 10 degrees with the derivative calculated during first four seconds of the occlusion. ROIs with distorted waveforms were marked as untrustworthy. All remaining ROIs were classified as “good”, and the speed of the PPG-waveform in these ROIs was calculated as the mean derivative of the signal during first four seconds after starting the occlusion. PPG-waveform speed was measured in percent per seconds. Figures 3 and 4 show examples of the spatial distribution of the position of “good” and untrustworthy ROIs in the forehand. To show dependencies of the PPG-waveform speed on the longitudinal and circumferential directions, the data from “good” ROIs were grouped in rows and columns, respectively. Examples of the circumferential distribution of the PPG-waveform speed is shown in Fig. 5a and c, whereas the speed dependencies along the forearm are shown in Fig. 5b and d.
